# Long-term livestock exclusion facilitates native woody plant encroachment in a sandy semiarid rangeland

**DOI:** 10.1002/ece3.1531

**Published:** 2015-05-25

**Authors:** Hua Su, Wei Liu, Hong Xu, Zongshuai Wang, Huifang Zhang, Haixiao Hu, Yonggeng Li

**Affiliations:** 1 State Key Laboratory of Vegetation and Environmental Change, Institute of Botany, Chinese Academy of SciencesBeijing, 100093, China; 2 National Engineering and Technology Center for Information Agriculture Key Laboratory of Crop Physiology and Ecology in Southern China, Ministry of Agriculture, Nanjing Agricultural UniversityNanjing, Jiangsu Province, 210095, China; 3 University of Chinese Academy of SciencesBeijing, 100049, China

**Keywords:** Fencing, Hunshandake, livestock exclusion, livestock grazing, semiarid rangeland, *Ulmus pumila*, woody plant encroachment

## Abstract

1. The role of livestock grazing in regulating woody cover and biomass in grass-dominant systems is well recognized. However, the way in which woody plant populations in respond when livestock are removed from grazing in the absence of other disturbances, such as fire, remains unclear.
2. We conducted a 10-year, replicated fencing experiment in a sandy semiarid rangeland in northern China (which has a mean annual rainfall of 365 mm), where fires have been actively suppressed for decades.
3. Fencing dramatically influenced the growth and age structure of the native tree species, *Ulmus pumila*, which is the sole dominant tree in the area. After a decade, the density of the *U. pumila* tree population in the fencing plots increased doubly and canopy cover increased triply. The proportion of both saplings (*U*_*2*_) and young trees (*U*_*3*_) increased in fencing plots but decreased in grazing plots after the 10-year treatment period. The effects of fencing on *U. pumila* trees varied by age class, with potential implications for the future structure of the *U. pumila* tree community. Decadal fencing led to approximately 80-fold increase in recruitment and a nearly 2.5-fold decrease in the mortality of both *U*_*2*_ and *U*_*3*_. Further, livestock grazing generated a “browsing trap” to the recruitment of both *U*_*2*_ and *U*_*3*_, and had a small impact on the mortality of old trees. A long-term, fencing-driven shift in woody species composition was mediated via its effects on both recruitment and mortality rates.
4. *Synthesis and applications*. Our results demonstrate that in the long-term absence of both fire and livestock, native woody plant encroachment tends to occur in sandy rangelands, transforming the woody plant demography in the process. The feasibility of full livestock exclusion in sandy rangelands requires further discussion. A balanced amount of livestock grazing may provide critical ecosystem services by regulating woody cover and mediating woody plant encroachment.

## Introduction

Increased woody plant abundance (encroachment) is occurring in rangelands worldwide (Roques et al. 2001; Rundel et al. 2014). Rangelands constitute approximately 50% of the earth's land surface and contain more than 30% of the world's human population (Anadón et al. 2014). These rangelands carry profound consequences for community structure, biodiversity and the functioning of rangeland ecosystems (Huxman et al. 2005; Hu et al. 2008; Richardson and Rejmánek 2011; Huang et al. 2012; Ratajczak et al. 2012). For example, the encroachment of woody plants into rangelands can alter the soil moisture (Pressland 1973) and nutrient and microclimate conditions (Belsky 1992), suppress grass productivity associated with animal production and wildlife conservation (Stuart-Hill and Tainton 1989; Hudak 1999; Dalle et al. 2006; Belay et al. 2013), and reduce stream flow and groundwater recharge (Archer 2010). It is necessary to study the factors that can cause woody plant encroachment to understand rangeland ecology and management (Holdo et al. 2009; Gordijn et al. 2012).

Woody plant encroachment appears to be caused by a combination of climate change, livestock grazing, fire suppression, and an increase in atmospheric CO_2_ (Archer et al. 1995; Morgan et al. 2007; Wigley et al. 2010; Eldridge et al. 2011; Buitenwerf et al. 2012; Kulmatiski and Beard 2013). Water availability plays a critical role in regulating tree cover and biomass in rangelands by effectively providing a climatic limit to the tree biomass (Park et al. 2012; Ratajczak et al. 2012; Su et al. 2014). However, “top-down” forces, such as herbivores and fire, also exert significant effects on tree cover and biomass and can profoundly alter the structure and composition of many rangeland ecosystems (Sankaran et al. 2013). Fires often limit tree cover and biomass by preventing seedling recruitment and sapling maturation to adults rather than directly killing adult trees (Hoffmann 1999; Higgins et al. 2000; Staver et al. 2009; Hean and Ward 2012). Fires are incapable of suppressing woodland regeneration (Holdo 2007). Fire and browsing, whose effects on vegetation are somewhat analogous (Bond 2005, 2008), must act synergistically to prevent increases in tree cover (Cabral et al. 2003; Holdo et al. 2013). However, in contrast to fire, the effects of herbivores on woody encroachment are difficult to generalize across rangeland ecosystems (Augustine and McNaughton 1998; Staver et al. 2009; Sankaran et al. 2013).

Livestock grazing, which is the most common form of land use in the world (Foley et al. 2005), has grown by 600% over the last three decades (Asner et al. 2004). Livestock grazing leads to woody encroachment once the stocking rate exceeds a threshold determined by the long-term mean annual rainfall (Jeltsch et al. 1997). It is widely accepted that overgrazing has facilitated woody plant invasion through a combination of factors (Jeltsch et al. 1997; Roques et al. 2001; Kambatuku et al. 2011), such as reducing competition from grasses (Auken 2000), dispersal of seeds of woody plants (Auken 2000; Tews et al. 2006), and changing rodent and insect populations (Jeltsch et al. 1997; Auken 2000; Roques et al. 2001; Kambatuku et al. 2011). However, browsing pressure can reduce the establishment of woody seedlings (Prins and van der Jeugd 1993) and retard the growth of woody plants (Pellew 1983). In semiarid rangelands, native browsing ungulates influence the recruitment, growth, and mortality of woody vegetation, and they consequently regulate woody cover. The removal of native browsing ungulates can lead to rapid woody encroachment (Sankaran et al. 2013). For example, Maher et al. (2010) found that an absence of kangaroos in fenced plots in kwongan of southwestern Australia would result in the much more extensive and rapid encroachment of *Allocasuarina huegeliana* than it would in unfenced plots. Livestock removal is often considered an essential tool for vegetation restoration and conservation in arid and semiarid rangelands (Frank et al. 2014). However, little is known about how livestock removal affects woody encroachment because isolating the effects of browsing on rangeland woody plant dynamics requires long-term, controlled livestock exclusion experiments, which are rare in Eurasian rangelands (Wesche and Treiber 2012).

We conducted a decade-long, replicated livestock exclusion experiment (fencing) with normal livestock grazing (1.4 sheep unit per ha) as a control in semiarid Hunshandake Sandy Land in northern China, where fires have been actively suppressed for decades. The site is savanna-like (Liu et al. 2013; Su et al. 2014) and is characterized as a continuous grass layer with scattered trees solely dominated by *Ulmus pumila* trees (Wallis de Vries et al. 1996; Solla et al. 2005; Jiang et al. 2006; Tang et al. 2014). Our specific objectives were to (1) quantify the long-term effects of fencing on *U. pumila* tree population dynamics in the absence of external perturbations, such as fire; and (2) determine whether fencing affects the recruitment and mortality of *U. pumila* trees.

## Materials and Methods

### Study site

The study was conducted at the Hunshandake Sandy Land Ecological Research Station (41°54′N, 116°0′E) of the Institute of Botany, Chinese Academy of Science in Inner Mongolia, northern China. The elevation is approximately 1300 m. The soil is composed primarily of eolian sands, which are often distributed in a mosaic pattern with sandy dark chestnut soil (Xu and Zou 1998). Meteorological data obtained from a weather station located in Zhenglanqi County (approximately 100 km from the site) indicate that the mean annual rainfall is 250–450 mm, with an average 367.1 mm for the period 2002–2012. Approximately 70% of the yearly rainfall events occur between June and August. The annual mean temperature is −1.7°C, with the lowest monthly mean value of −23.4°C in January and the highest monthly mean value of 14.7°C in July. The average frost-free period lasts approximately 110 days. Vegetation in the area is characterized as a savanna-like woodland community (Liu et al. 2013; Su et al. 2014) and a discontinuous layer of perennial grasses (Jiang et al. 2006). *Ulmus pumila*, the sole dominant tree species, is distributed sparsely on the fixed dunes, typically with an open canopy structure (Su et al. 2014). The shrub layer is dominated by *Salix gordejevii*,*S. microstachya,* and *Caragana microphylla* (Su et al. 2009). The herbaceous layers are dominated by grassland plants that are common to Eurasian steppe zone, including *Leymus chinensis*,*Agropyron cristatum,* and the forb *Artemisia frigida*.

The most common livestock at the study zone are cattle, sheep, and camel (Ding et al. 2005). By the 1980s, the national government had encouraged nomadic herders to move to villages and increase their herds to boost incomes. The average stocking rate reached approximately 2.6 sheep unit ha^−1^, a rate that is much greater than the theoretical stocking rate (1.2–1.8 sheep unit ha^−1^) (Ding et al. 2005). Year-long continuous livestock grazing-induced directional shift degraded most of the available rangelands by 2000 (Ding et al. 2005; Zheng et al. 2006). A demonstration project was launched in 2001 that excluded livestock from 2670 hectares of communal rangeland.

### Field investigations

A large enclosure (2670 ha) protected with 1.5-m-tall wire mesh fences was established at the study site in 2001. To ensure the exclusion of all domestic animals, the local government hired people to conduct daily checks. Meanwhile, the remaining rangeland (approximately 5000 ha) was used for livestock grazing at a stocking rate of 1.4 ± 0.4 sheep unit per ha. Five plots (100 × 100 m) with similar landscapes and soil conditions were selected in the aforementioned fencing and grazing sites. We mapped the individual *U. pumil*a trees in each fencing and grazing plot and measured their density, basal area (at 15 cm aboveground level), diameter, height, aboveground biomass, and canopy cover (the proportion of the floor covered by the vertical projection of the tree crowns).

In 2002, we measured the base diameter (*d*) and height of all trees in plots using a diameter tape. The diameter at breast height (*dbh*) of trees higher than 1.5 m was also measured. *dbh* is a strong indicator of tree biomass (Zianis 2008). The aboveground biomass (AGB) of the *U. pumila* trees in the plots was estimated based on the allometric equation, where *AGB* = *0.1196 dbh*^*2.2201*^ (*R*^2^ = 0.9968, bias error = 5.61%). To develop the above allometric equation, fifty individual trees with different sizes were randomly selected and destructively sampled during their most vigorous growth period (August). The aboveground portion of each tree was air-dried to a constant weight and weighed on a large platform scale (Yaohua Co. Ltd., Shanghai, China) in the field (±10 g) for AGB. The AGB data were then analyzed using an allometric equation following that of Kuyah et al. (2012). All of the AGB data for the other *U. pumila* trees (height ≤1.5 m) were obtained through actual measurements. Canopy cover was ocular and estimated by three experienced forestry professionals (Korhonen et al. 2006). The total canopy cover of large trees (height >1.5 m) was recorded.

In 2012, all of the measurements except the canopy cover measurements were retaken. We then estimated the canopy cover using digital photographs that were obtained by the methods described by Korhonen et al. (2006). The original images were converted into binary images using *ImageJ* image analysis software (1.42q, National Institutes of Health, Bethesda, MD). Crowns in binary images were painted black such that the result would be closer to the traditional canopy cover estimate.

To better understand fencing effects on *U. pumila* tree population dynamics in this system, changes in the biomass, basal area, height, recruitment, and mortality were analyzed separately for each age class: seedlings (*U*_*1*_, *d *<* *0.5 cm), saplings (*U*_*2*_, 0.5 ≤ *d *<* *5 cm), young trees (*U*_*3*_, 5 ≤ *d *<* *20 cm), mature trees (*U*_*4*_, 20 ≤ *d *<* *40 cm), and old trees (*U*_*5*_, *d *>* *40 cm). The performance of each age class is presented in Table1. To compare the population structure, the proportion of each age class relative to the *U. pumila* tree populations in grazing and fencing plots was calculated over the 10-year treatment period. We calculated the decadal changes in AGB, basal area, height, recruitment, and mortality at each age class level over the 10-year treatment period. Recruitment here refers to all new individuals of each age class recorded in the 2012 census but not in the 2002 census. Recruitment is thus an integrated measure of effective recruitment over a 10-year period and does not include data on individuals that were recruited and died within that period. Mortality similarly refers to the fraction of all living individuals in each age class that were present in the 2002 census but died by the 2012 census.

**Table 1:** Performance of seedlings (*U*_*1*_), saplings (*U*_*2*_), young (*U*_*3*_), mature (*U*_*4*_), and old (*U*_*5*_) *Ulmus pumila* trees in Hunshandake Sandy Land

Class	Age (yr)	Diameter (cm)	Height (m)
*U*_*1*_	<2	<0.5	<0.5
*U*_*2*_	2–10	0.5–5	0.5–2
*U*_*3*_	10–30	5–20	2–5
*U*_*4*_	31–50	20–40	5–8
*U*_*5*_	>50	>40	>8

We evaluated the effects of fencing on the survival rates of *U*_*1*_ (divided into the current year and a 2-year period) and *U*_*2*_. We counted the number of *U*_*1*_ and *U*_*2*_ individuals in the grazing and fencing plots (*n*_*0*_) in May 2002. After 3 months, the living individuals in the plots (*n*_*a*_) were counted again, and the survival rate was calculated as follows: survival rate (%) = 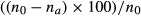. We repeated the measurements in 2004, 2006, and 2008.

### Statistical analysis

A two-way repeated-measures analysis of variance (ANOVA) was employed to detect the significance of canopy cover, AGB, tree density, and basal area of the *U. pumila* tree population at the whole-plot level and in each age class, using treatment (fencing and grazing) and year, and their interactions as fixed factors. We used paired *t*-tests to evaluate the effects of fencing on the decadal recruitment and decadal mortality of the *U. pumila* tree population between 2002 and 2012. The statistical analyses were performed using SPSS version 18.0 (SPSS Inc., Chicago, IL). A post hoc least significant difference (LSD) test was implemented in *P *=* *0.05 (significant at *P *<* *0.05).

## Results

### Responses of total *Ulmus pumila* tree population

**Figure 1:**
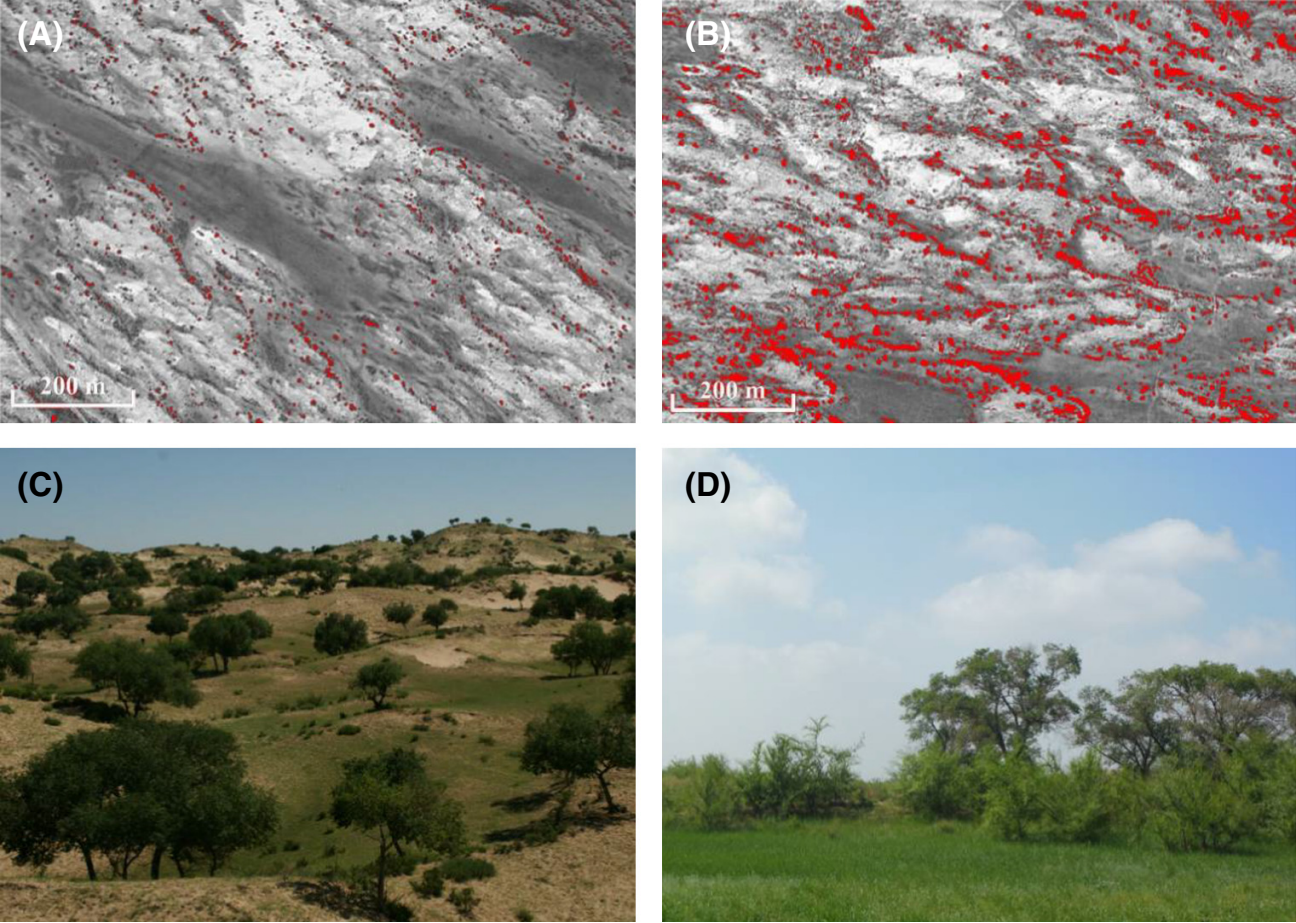
Satellite images show the distribution of *Ulmus pumila* trees (trees whose canopy area is larger than 2 m^2^ were painted red) in grazing (A) and fencing (B) plots. Field photos show the generational status of *U. pumila* trees in grazing (C) and fencing (D) plots. The background images were created by Google Earth.

**Figure 2:**
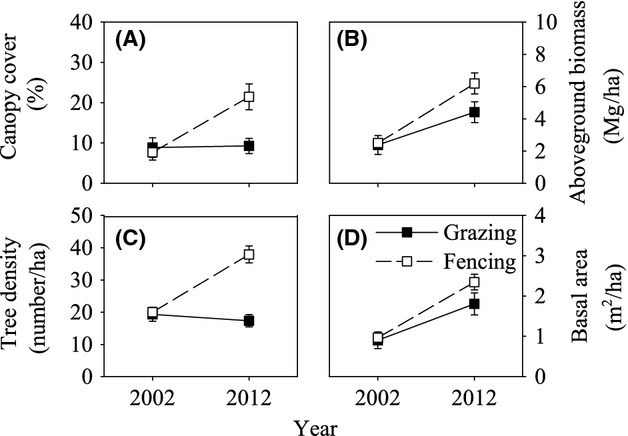
Decadal changes in canopy cover (A), total aboveground biomass (B), tree density (C), and total basal area (D) of *Ulmus pumila* trees in grazing and fencing plot. Basal area and biomass values have been scaled up from the plot level (100 × 100 m) and are reported on a per hectare basis. Error bars represent ±SE.

*Ulmus pumila* is nearly the sole dominant native tree species growing on the study site (Liu et al. 2013; Su et al. 2014). The decade-long fencing dramatically influenced the *U. pumila* tree population attributes in plots (Figs.1, 2). Woody plant encroachment substantially increased in the fencing plots during the 10-year period (Fig.1B). Long-term fencing significantly changed the canopy cover (*P *<* *0.05) and tree density (*P *<* *0.05) (Table2). In fencing plots, the total canopy cover of the *U. pumila* tree population rose significantly (*P *<* *0.05), from 9.2% in 2002 to 24.7% in 2012, a nearly threefold increase over the 10-year treatment period (Fig.2A). The total density of *U. pumila* trees doubled (*P *<* *0.05), from 20.03 individuals per ha in 2002 to 37.93 individuals per ha in 2012 (Fig.2C). The effects of fencing on the aboveground biomass and basal area of the *U. pumila* trees were slight and insignificant (*P *>* *0.05). Time has significant effect on the canopy cover, tree density, aboveground biomass, and basal area of the *U. pumila* tree population (*P *<* *0.05). Both aboveground biomass and basal area increased more than 2 times from 2002 to 2012 (Fig.2B and D).

**Table 2:** Results of two-way repeated-measures ANOVA testing the effects of treatment (fencing and grazing) and time (before and after) on canopy cover, aboveground biomass, tree density, and basal area of the studied *Ulmus pumila* trees

Variables	Canopy cover		Aboveground biomass		Tree density		Basal area	
	*F*	*P*	*F*	*P*	*F*	*P*	*F*	*P*
Treatment	23.75	0.02*	1.46	0.31^ns^	35.25	0.01*	1.08	0.38^ns^
Time	73.95	0.00*	17.11	0.03*	80.73	0.00*	60.95	0.00*
Treatment × Time	15.49	0.03*	1.20	0.35^ns^	50.69	0.01*	0.52	0.52^ns^

*F*-test values and *P*-values are given.

* represents *P *<* *0.05 and ^ns^represents *P *>* *0.05.

**Figure 3:**
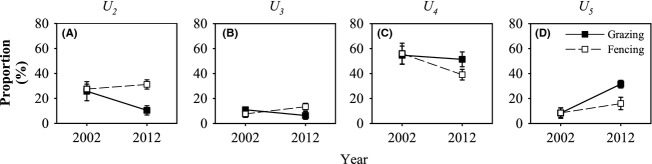
Decadal changes in the proportion of each age class relative to total *Ulmus pumila* trees in grazing and fencing plots. *U*_*2*_: *U. pumila* saplings, *U*_*3*_: young *U. pumila* tree, *U*_*4*_: mature *U. pumila* tree, *U*_*5*_: old *U. pumila* tree. Error bars represent ±SE.

Decadal fencing altered the age structure of the *U. pumila* trees, the proportion of each age class was significantly changed (*P *<* *0.05) (Table3). The proportion of saplings (*U*_*2*_) and young trees (*U*_*3*_) in fencing plots significantly increased (*P *<* *0.05), accounting for approximately 31.2% and 13.5%, respectively, in 2012, higher than that in 2002 by 13.3% and 74.5%, respectively (Fig.3A and B). By contrast, the proportion of *U*_*2*_ and *U*_*3*_ in grazing plots declined over the 10-year treatment period, from 25.8% and 11.0%, respectively, in 2002 to 10.5% and 6.5%, respectively, in 2012 (*P *<* *0.05). The reduction achieved was 59.3% and 41.0%, respectively (Fig.3A and B). Fencing had no significant effects on mature *U. pumila* trees (*U*_*4*_) and old *U. pumila* trees (*U*_*5*_) (*P *>* *0.05) (Table3). The proportion of *U*_*4*_ decreased in both the grazing and fencing plots (Fig.3C), and the proportion of *U*_*5*_ climbed in both grazing and fencing plots (Fig.3D).

**Table 3:** Results of two-way repeated-measures ANOVA testing the effects of treatment (fencing and grazing) and time (before and after) on the proportion of saplings (*U*_*2*_), young (*U*_*3*_), mature (*U*_*4*_), and old (*U*_*5*_) *Ulmus pumila* trees

Variables	*U*_*2*_		*U*_*3*_		*U*_*4*_		*U*_*5*_	
	*F*	*P*	*F*	*P*	*F*	*P*	*F*	*P*
Treatment	27.42	0.01*	12.05	0.04*	0.02	0.88^ns^	2.28	0.23^ns^
Time	9.91	0.05*	2.91	0.19^ns^	1.06	0.38^ns^	18.72	0.02*
Treatment × Time	15.74	0.03*	35.91	0.01*	12.23	0.04*	11.64	0.04*

*F*-test values and *P*-values are given.

* represents *P *<* *0.05 and ^ns^represents *P *>* *0.05.

### Responses of *Ulmus pumila* trees in different age classes

**Figure 4:**
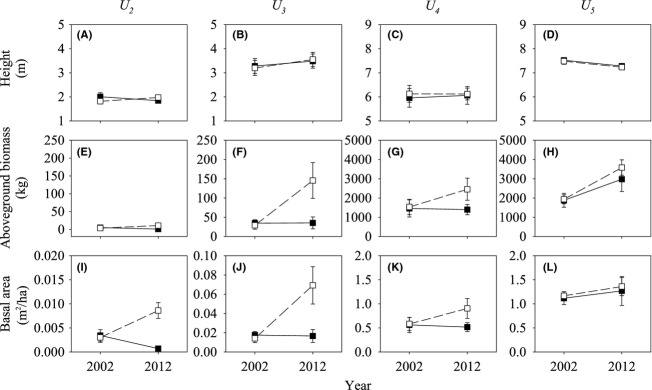
Decadal changes in aboveground biomass (A), height (B), and basal area (C) of saplings (*U*_*2*_), young (*U*_*3*_), mature (*U*_*4*_), and old (*U*_*5*_) *Ulmus pumila* trees in grazing and fencing plots. Basal area and biomass values have been scaled up from the plot level (100 × 100 m) and are reported on a per hectare basis. Error bars represent ±SE.

Fencing was beneficial for the height increment of *U. pumila* trees except for *U*_*5*_ (Fig.4A–D). *U*_*2*_ and *U*_*3*_ changed significantly in response to grazing and fencing, and *U*_*4*_ and *U*_*5*_ changed insignificantly (*P *<* *0.05) (Table4). In the absence of livestock, the height of *U*_*2*_ and *U*_*3*_ increased from 15.84 ± 6.44 cm and 35.11 ± 30.14 cm, respectively. By contrast, livestock grazing significantly reduced the height of *U*_*2*_ and *U*_*3*_, from 16.16 ± 9.08 cm and 20.46 ± 9.32 cm, respectively. In the absence of livestock, *U. pumila* trees in each age class showed substantial increases in aboveground biomass over the 10-year period (Fig.4E–F). However, fencing had significant effects on both *U*_*2*_ and *U*_*3*_ (*P *<* *0.05), and insignificant effects on both *U*_*4*_ and *U*_*5*_ (*P *>* *0.05) (Table4). The aboveground biomass of different age classes showed significant different response magnitudes to fencing. *U*_*3*_ increased the most (Fig.4F), with a value of 229.84 ± 46.47 kg ha^−1^, followed by *U*_*4*_, *U*_*5*_, and *U*_*2*_. We found no net changes in the aboveground biomass of *U*_*2*_ and *U*_*3*_ in grazing plots over the 10-year period, unlike *U*_*4*_ and *U*_*5*_, whose aboveground biomass increased even in the presence of livestock grazing (Fig.4G and H). Similar to the patterns observed for aboveground biomass, fencing had significant effects on the basal area of both *U*_*2*_ and *U*_*3*_ (*P *<* *0.05) and insignificant effects on both *U*_*4*_ and *U*_*5*_ (*P *>* *0.05) (Table4). In the fencing plots, decadal changes were the largest in the basal area of *U*_*5*_, with a value of 9918.14 ± 188.32 cm^2^ ha^−1^, followed by *U*_*4*_ (3242.35 ± 70.25 cm^2^ ha^−1^) and *U*_*3*_ (550.56 ± 193.80 cm^2^ ha^−1^). Decadal changes in the basal area of *U*_*2*_ were undetectable. There were no detectable changes in the basal area of all four age classes in grazing plots over the 10-year period.

**Table 4:** Results of two-way repeated-measures ANOVA testing the effects of treatment (fencing and grazing) and time (before and after) on the height, aboveground biomass, and basal area of saplings (*U*_*2*_), young (*U*_*3*_), mature (*U*_*4*_), and old (*U*_*5*_) *Ulmus pumila* trees

	Variables	*U*_*2*_		*U*_*3*_		*U*_*4*_		*U*_*5*_	
		*F*	*P*	*F*	*P*	*F*	*P*	*F*	*P*
Height	Treatment	5.07	0.01*	5.63	0.09*	0.07	0.80^ns^	0.04	0.85^ns^
	Time	0.00	0.99^ns^	0.24	0.66^ns^	0.01	0.93^ns^	3.83	0.15^ns^
	Treatment × Time	0.33	0.6^ns^	0.04	0.86^ns^	0.02	0.89^ns^	0.00	0.98^ns^
Aboveground biomass	Treatment	7.65	0.07*	8.51	0.06*	1.08	0.38^ns^	0.41	0.57^ns^
	Time	1.05	0.38^ns^	1.44	0.32^ns^	0.76	0.45^ns^	27.47	0.01*
	Treatment × Time	2.26	0.23^ns^	2.75	0.20^ns^	0.95	0.40^ns^	0.57	0.50^ns^
Basal area	Treatment	11.23	0.04*	12.70	0.04*	1.18	0.36^ns^	0.07	0.81^ns^
	Time	1.71	0.28^ns^	1.77	0.28^ns^	0.77	0.44^ns^	37.66	0.01*
	Treatment × Time	2.52	0.21^ns^	3.43	0.16^ns^	1.27	0.34^ns^	0.01	0.94^ns^

*F*-test values and *P*-values are given.

* represents *P *<* *0.05 and ^ns^represents *P *>* *0.05.

**Figure 5:**
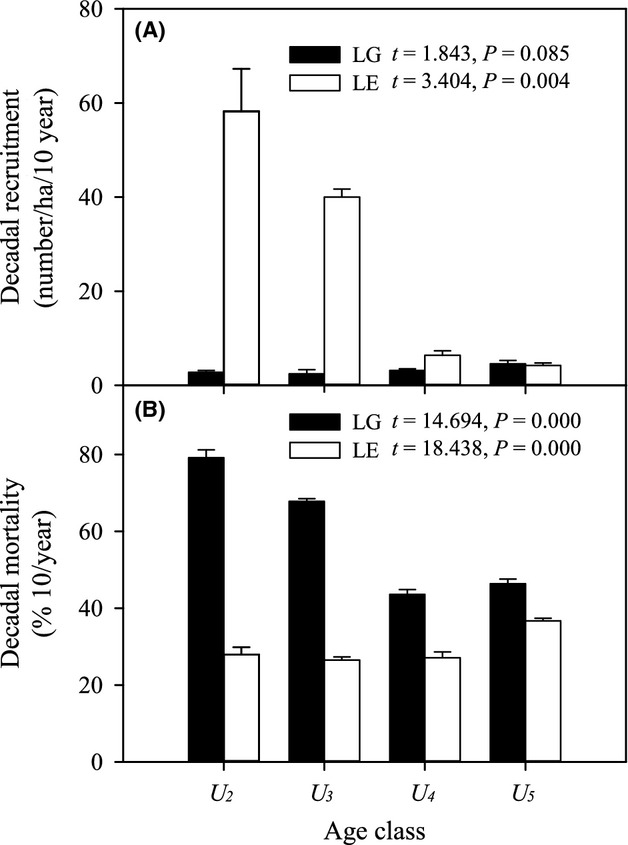
Decadal recruitment (A) and decadal mortality (B) of saplings (*U*_*2*_), young (*U*_*3*_), mature (*U*_*4*_), and old (*U*_*5*_) *Ulmus pumila* trees in grazing and fencing plots. Estimate of recruitment has been scaled up from the plot level (100 × 100 m) and reported on a per hectare basis. Error bars represent ±SE.

All age classes except *U*_*5*_ showed significantly higher recruitment in fencing plots than in grazing plots (Fig.5A). Compared with grazing, the recruitment of both *U*_*2*_ and *U*_*3*_ increased approximately 80-fold in the fencing plots (*P *<* *0.05). Unlike the pattern observed for recruitment, significant changes in decadal mortality were found in both grazing plots and fencing plots (*P *<* *0.05) (Fig.5B). Protection from livestock dramatically reduced mortality rates for *U*_*2*_, *U*_*3*_, and *U*_*4*_, but not for *U*_*5*_ (Fig.5B). *U*_*2*_ had the highest mortality in grazing plots, at approximately 79.2%, followed by *U*_*3*_, with a value of approximately 67.8%. In the absence of livestock, the mortality rates did not differ significantly among *U*_*2*_, *U*_*3*_, and *U*_*4*_, which averaged approximately 27.2% per decade (Fig.5B). *U*_*5*_ had the highest mortality rate (∼36.7%) in fencing plots, but nearly the lowest (∼46.4%) in grazing plots.

### Responses of generation recruitment of *Ulmus pumila* tree

**Figure 6:**
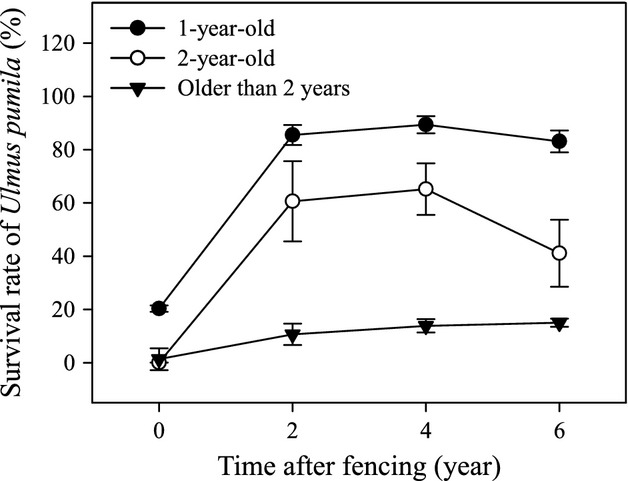
The effect of grazing and fencing on the survival rates of current-year and 2-year-old seedlings (*U*_*1*_) and *U. pumila* saplings (*U*_*2*_). Error bars represent ±SE.

Fencing significantly increased the survival rates of *U. pumila* seedlings and saplings (Fig.6). For example, after fencing for 2 years, the survival rate of current-year, 2-year, and older *U. pumila* were 10.7%, 60.6%, and 85.5%, respectively, which were much higher than the survival rates prior to fencing (similar to grazing plots) (Fig.6). However, there were no meaningful differences among the survival rates after excluding livestock for a longer period of time, regardless of whether the trees were seedlings or saplings. Livestock grazing controlled generation recruitment effectively, with almost no seedlings surviving prior to fencing (Fig.6). However, the survival rate of saplings was approximately 20.3%, even in grazing plots (Fig.6).

## Discussion

*Ulmus pumila* is widely distributed in the north temperate zone (Solla et al. 2005) and contributes to a special savanna-like woodland in the rangelands of northern China (Liu et al. 2013; Su et al. 2014) and Mongolia (Dulamsuren et al. 2009a,b). Dulamsuren et al. (2009a) presumed that *U. pumila* cannot encroach onto the south-facing slopes covered with steppe and savanna-like *U. pumila* woodland, even without livestock grazing. Nonetheless, the results from our decade-long fencing experiment indicate that livestock exclusion is capable of inducing *U. pumila* encroachment in the Hunshandake sandy rangeland in northern China, thereby dramatically altering the structure and function of native dominant trees. The decade-long livestock exclusion not only resulted in a double to triple increase in the canopy cover and density of the *U. pumila* tree population but also led to approximately an 80-fold increase in recruitment and a nearly 2.5-fold decrease in mortality in both saplings (*U*_*2*_) and young trees (*U*_*3*_) in this ecosystem.

The large increment of total *U. pumila* tree biomass that was observed in fencing plots (Fig.2B) is in accordance with the results of Riedel et al. (2013), who found that woody plant biomass in Mediterranean woodland doubled in undisturbed, nongrazed areas. However, the results are attributable to significant tree growth over time and not to fencing (Table2). The abundance of native tree species increased significantly following the exclusion of browsing herbivores (Fig.2C and Table2). Coincidentally, the same phenomenon has been observed in temperate savanna (Weltzin et al. 1997), temperate grassy woodland (Kirkpatrick 2004), grassland (Noble et al. 2007), and other areas. However, the fact that total tree biomass also increased in grazing plots, although to a lesser extent than in fencing plots (Fig.2B), indicates that the current grazing regimes presented here (1.4 sheep unit ha^−1^) were able to modulate but not stop the succession of tree vegetation toward the climax vegetation types, which would consist of *U. pumila* forest or *Pinus sylvestris* var. *mongolica* forest (Liu et al. 2009a). The results obtained from Mediterranean areas (Casasús et al. 2007; Riedel et al. 2013), whose landscapes are similar to our study site, show that livestock grazing at moderate stocking rates can halt woody plant encroachment.

Our results indicate that the effects of fencing on *U. pumila* trees varied by age classes, with potential implications for the future structure of the *U. pumila* tree community. In particular, fencing had exerted a significant influence on the dynamics of *U*_*2*_ and *U*_*3*_ compared with other age classes (Table3 and 4, Figs.3, 4). The proportion of *U*_*2*_ and *U*_*3*_ climbed by 13.3% and 74.5%, respectively, after the 10-year fencing (Fig.3). Livestock grazing significantly influenced the recruitment of *U*_*2*_ and *U*_*3*_ (Fig.5A) and had virtually no impact on the mortality of *U*_*4*_ and *U*_*5*_ (Fig.5B). Consequently, only *U*_*5*_ showed a net increase in basal area and biomass in grazing plots over the decade (Fig.4), which suggests a long-term livestock grazing-induced directional shift in the *U. pumila* age structure toward aging. Our results are consistent with similar long-term experiments that were conducted in the semiarid savanna (Sankaran et al. 2013). Year-round chronic browsing by domestic livestock constrains woody biomass by generating a “browsing trap,” particularly for *U*_*2*_ and *U*_*3*_. Significant differences were found in the structure of *U. pumila* trees between fencing and grazing plots after decadal treatment (Fig.3 and Table3). At the beginning of our study, *U*_*4*_ occupied the largest proportion (approximately 55%) of the site. However, the proportion of *U*_*2*_, *U*_*3*_, and *U*_*5*_ increased after a decade of fencing, and only *U*_*5*_ increased in the grazing plots. The recruitment of both *U*_*2*_ and *U*_*3*_ (0.8 ± 0.04 and 0.5 ± 0.09 individual ha^−1^ year^−1^) was substantially controlled by livestock grazing. The results may indicate that livestock grazing can easily control young trees but not adult trees because of the latter's large size (Archibald and Bond 2003; Ward and Esler 2011; Hean and Ward 2012). However, herbivores did not cause lethal damage in *U. pumila* trees beyond the age of seedlings (Dulamsuren et al. 2009a), but cause stunting and twisting in mature *U. pumila* trees (Liu et al. 2013). Domestic livestock (e.g., cattle and sheep) prefer young leaves, which have higher nitrogen and water levels, to mature leaves (Coley and Barone 1996). Between 5% and 10% of the leaf area of *U. pumila* trees was consumed by herbivores (Dulamsuren et al. 2009a), which is sufficient to reduce plant fitness and substantially impact the growth and survival of plants (Coley and Barone 1996), consequently depressing the survival of seedlings and small trees. We did not quantify the indirect effects of livestock, such as those arising from changes in grass biomass and the strength of tree–grass competition (Riginos and Young 2007; Riginos 2009), as well as the abundance of other cryptic consumers, such as rodents, which can be important to tree survival in some ecosystems (Goheen et al. 2004; Maclean et al. 2011). Although future studies would benefit from a quantification of both the direct and indirect effects of different age classes of trees, our results suggest that the direct effects of livestock, particularly on sapling and young trees, are substantial.

Our results support the opinion that *U. pumila* seedlings older than 2 years of age have increased survival rates (Dulamsuren et al. 2009b). Dulamsuren et al. (2009b) analyzed whether xeric microclimate and high herbivore densities limit the success of seedling establishment in *U. pumila* tree and found that most of the seedlings older than 2 years survived after suffering from feeding damage by insects and small mammals, from nitrogen deficiency, and, to a lesser degree, from drought. The survival rates of seedlings older than 2 years could exceed 20% even with livestock grazing (Fig.6). The germination of *U. pumila* seeds is not a problem in *U. pumila* open woodland (Guo and Liu 2004), but the high susceptibility of newly emerged seedlings to environmental stresses and disturbances is a serious bottle neck for *U. pumila* that prevents them from encroachment (Dulamsuren et al. 2009b). Livestock grazing considerably suppressed the survival of seedlings younger than 2 years of age and kept their survival rate below 5% (Fig.6). Livestock exclusion stimulates the outbreak of seedlings and protects more seedlings across their receptive stage successfully (Fig.6). The density of *U. pumila* seedlings can reach 288 per m^2^ in fenced areas (Liu et al. 2005), and their density is only 4 per m^2^ in heavily grazed sites (Li 2003). The survival rates can exceed 10% and 60% for the current-year and 2-year-old seedlings, respectively (Fig.6). This finding may be explained by the fact that *U. pumila*, as the dominant native species, produces a number of seeds that easily geminate in the local soil (Liu et al. 2009b). However, the survival of the seedlings can be affected by the presence of livestock through consumption and physical damage, such as trampling, which in turn can impact their growth and reproduction patterns (Cairns and Moen 2004).

Trees are typically long-lived, and tree dynamics in arid and semiarid areas often tend to be “event-driven,” where the timing and magnitude of episodic rainfall events drive plant growth, mortality, and other ecosystem processes (Sankaran et al. 2013). Consequently, general trends are difficult to show in short-term studies that are likely to miss significant recruitment or dieback events and thus incorrectly estimate transitions. Our findings show that livestock grazing maintains the woody layer in a low-density state dominated by *U*_*4*_ individuals and that fencing generates a high-density state dominated by younger *U*_*2*_ and *U*_*3*_ (Fig.3). These shifts occur not just because livestock exclusion substantially increases rates of recruitment and plant growth but also because over longer timescales, fencing reduces rates of mortality for older ones (Fig.5). Our results highlight the importance of long-term experimental studies for a detailed understanding of fencing or grazing effects on woody vegetation dynamics. A short-term effect may be misleading (Bakker 1998). For example, our prediction, which was made 3 years after fencing, was that natural restoration would be practicable without any artificial management (Normile 2007). However, after 10 years, our results indicate that artificial management is necessary to prevent woody plant encroachment. Modest livestock grazing appears to stabilize the functioning of these rangeland ecosystems (Bai et al. 2004).

**Figure 7:**
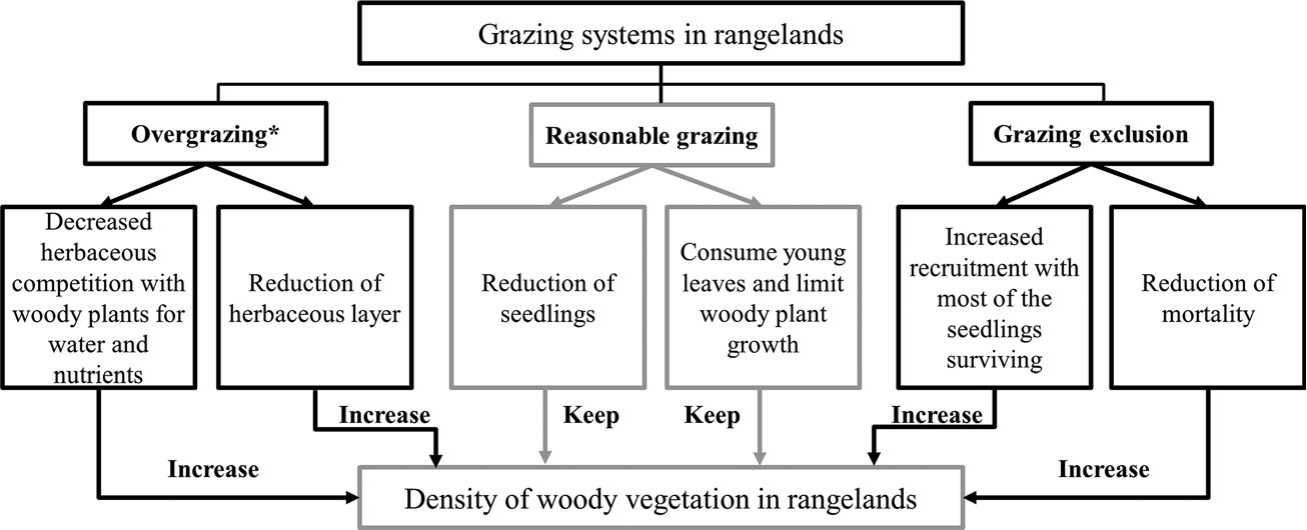
Processes mediating woody vegetation encroachment in different grazing systems. * refers to Asner and Martin (2004).

Reduced grazing pressure or even the disappearance of grazing livestock in some areas has been consistently reported, *for example*, Li and Huntsinger (2011) and Zhou et al. (2013) in China, which may have detrimental effects on sandy rangelands. However, woody plant encroachment does not necessarily occur after the cessation of grazing (Bakker 1998). The results may depend on the vegetation type, grazing regime, and the socioeconomic environment that is associated with livestock grazing systems in the study area. Grazing can control the dominance of certain plant species and enhance structural heterogeneity by selective defoliation, trampling, nutrient cycling, and propagule dispersal (Fig.7, Rook and Tallowin 2003). Therefore, the feasibility of full livestock exclusion requires further discussion. Before an expanding exclosure, the ecological consequences of additional exclosures should be investigated because long-term exclosures can increase woody plant encroachment.
